# A Novel Cell Type Enables *B. subtilis* to Escape from Unsuccessful Sporulation in Minimal Medium

**DOI:** 10.3389/fmicb.2016.01810

**Published:** 2016-11-11

**Authors:** Hervé Joël Defeu Soufo

**Affiliations:** Division of Infectious Diseases, University Medical Center FreiburgFreiburg, Germany

**Keywords:** sporulation, minimal growth medium, cell division, minicell, dwarf cell

## Abstract

Sporulation is the most enduring survival strategy developed by several bacterial species. However, spore development of the model organism *Bacillus subtilis* has mainly been studied by means of media or conditions optimized for the induction of sporogenesis. Here, I show that during prolonged growth during stationary phase in minimal medium, *B. subtilis* undergoes an asymmetric cell division that produces small and round-shaped, DNA containing cells. In contrast to wild-type cells, mutants harboring *spo0A* or *spoIIIE*/*sftA* double mutations neither sporulate nor produce this special cell type, providing evidence that the small round cells emerge from the abortion of endospore formation. In most cases observed, the small round cells arise in the presence of sigma H but absence of sigma F activity, different from cases of abortive sporulation described for rich media. These data suggest that in minimal media, many cells are able to initiate but fail to complete spore development, and therefore return to normal growth as rods. This work reveals that the continuation of asymmetric cell division, which results in the formation of the small round cells, is a way for cells to delay or escape from—unsuccessful—sporulation. Based on these findings, I suggest to name the here described cell type as “dwarf cells” to distinguish them from the well-known minicells observed in mutants defective in septum placement or proper chromosome partitioning.

## Introduction

In response to nutrient deprivation, some bacterial species within the Firmicutes phylum differentiate into dormant and non-reproductive cells called endospores. This highly resistant form of bacteria can survive harsh conditions that are usually lethal to their vegetative counterparts. The model organism, *Bacillus subtilis*, is to date the best-studied organism in terms of spore development. Numerous genes are up- or down-regulated or even switched on or off in the process of endospore formation. The process initially involves asymmetric cell division near one of the cell poles, which results in the formation of a smaller cell, the prespore or forespore, and a larger cell, the mother cell (stages I–II). The latter cell subsequently engulfs the forespore (stages II–III), ensures its proper development to the endospore [cortex synthesis (stage IV); spore coat synthesis and spore maturation (stages V–VI)], and finally undergoes autolysis releasing a mature spore (stage VII) (Errington, 1993, 2003; Stragier and Losick, 1996; Eijlander et al., 2014).

*B. subtilis* cells grown to stationary phase are heterogenic as they display a multitude of cell types associated with distinct differentiation pathways (Kearns and Losick, 2005). This heterogeneity is in part due to the bistable expression/activation (“ON” or “OFF”) states of regulatory proteins (Dubnau and Losick, 2006). In *B. subtilis*, the entry into sporulation is subject to bistability (Veening et al., 2005), which is regulated by the expression and phosphorylation level of the transcription factor and response regulator Spo0A~P (Hoch, 1993). Spore development is initiated when the cellular level of Spo0A~P reaches the threshold level (Fujita et al., 2005). Indeed, the level of Spo0A~P increases gradually as the cell enters the stationary phase (Fujita and Losick, 2005). The level of Spo0A~P is tightly regulated through a phosphorelay system. Environmental stimuli, cell cycle cues or metabolic factors trigger the autophosphorylation of KinA, the first kinase of the system. Thereafter, a flux of phosphate follows through the phosphorelay to the final acceptor Spo0A via intermediate kinases Spo0F and Spo0B, respectively (Burbulys et al., 1991; Grossman, 1991; Trach et al., 1991; Sonenshein, 2000). Sublevels of Spo0A~P are proposed to mediate the regulation of alternative developmental fates such as cannibalism and biofilm formation (Fujita and Losick, 2005). The decision of the cell to sporulate is thus the ultimate adaptive response to starvation. The success of the spore development resides in the compartmentalization of the gene expression established by two sigma factors σ^F^ in the forespore and σ^E^ in the mother cell (Errington and Illing, 1992). Both sigma factors become active only after the completion of the septum. This later event is reported to set the commitment to sporulation: The “point of no return” (Losick and Stragier, 1992; Parker et al., 1996; Dworkin and Losick, 2005). However, the activation of both σ^F^ and σ^E^ depends on subthreshold levels of Spo0A activity. At some subthreshold levels of Spo0A activity, cells that activate σ^F^ may fail to activate σ^E^ causing the abortion of sporulation, while most of the cells with active σ^E^ produce spores (Narula et al., 2012). This finding rather sets the σ^*E*^ activation as the ultimate sporulation decision point.

Most studies investigating spore development rely on three major protocols involving chemical induction (i.e., by the addition of decoyinine) (Grossman and Losick, 1988), resuspension method (Sterlini and Mandelstam, 1969) or the exhaustion in Difco sporulation medium (DSM) (Schaeffer et al., 1965b). These methods aim at maximizing sporulation efficiency and therefore do only poorly reflect sporulation under undisturbed growth conditions. Because of this experimental bias, only little is known on the sporulation behavior of wild-type *B. subtilis* grown in standard growth media. To address this issue, I set out to investigate stationary phase cells, which were grown in S7_50_ minimal medium, conventionally used for *B. subtilis* lab cultures.

In this medium, spore development is not an intrinsically robust process since many cells do not follow this pathway after asymmetric division. Instead, the unequally sized cells separate by generating an unusually small round and a normal cell. These findings reveal that the formation of small round cells by spore forming bacteria could provide an escape route from unsuccessful sporulation, or, alternatively, provide a strategy to avoid the costly spore development process under minimal medium growth condition.

## Materials and methods

### Growth conditions

Strains were grown at room temperature (25°C) with shaking at 200 rpm in Lysogeny Broth (LB) (Bertani, 2004), DSM (Schaeffer et al., 1965b), or S7_50_ minimal medium containing 50 mM MOPS (adjusted to pH 7.0 with KOH), 10 mM (NH_4_)_2_SO_4_, 5 mM potassium phosphate (pH 7.0), 2 mM MgCl_2_, 0.7 mM CaCl_2_, 50 μM MnCl_2_, 1 μM ZnCl_2_, 1 μg/ml thiamine-HCl, 20 μM HCl, 5 μM FeCl_3_ and 1% glucose supplemented with 0.1% glutamate and 0.004% casamino acids (Grossman and Losick, 1988; Harwood and Cutting, 1990). Spectinomycin (100 μg/ml), chloramphenicol (5 μg/ml), macrolide-lincosamide-streptogramin (MLS) (1 μg/ml erythromycin and 25 μg/ml lincomycin), or kanamycin (10 μg/ml) were added to the growth medium where appropriate. The growth medium was supplemented with 1 μM IPTG when required. For comparative analysis of growth, small round cells formation and sporulation, samples were collected at regular time intervals for OD_600_ measurement and evaluation of the % of small round cells and sporulation. The % of small round cells was determined by cell counts on acquired images. The evaluation of the % of sporulation is described below (see the “heat kill assay” method). Unless stated otherwise, samples were collected after 90–100 h of growth for microscopy. To investigate the viability of small round cells, cultures of *B. subtilis* generating small round cells were diluted 1:10 into fresh growth medium and grown for 60 min prior to the time-lapse experiments. Table 1 lists all the strains used in this work.

**Table 1:** **Strains**.

**Strain**	**Genotype**	**Source or references**
PY79	Prototrophic *of Bacillus subtilis* strain 168 (*trpC2*) derivative	Webb et al., 1997
NCIB 3610	Undomesticated *B. subtilis* strain	Bacillus Genetic Stock Center (BGSC)
FG493/1920	*minD::ermC divIVA::tet*	Marston and Errington, 1999
MF248	*amyE::P_*spoIIG*_-gfp spc*	Fujita and Losick, 2003
PY180 (IB206)	*spoIIE::*Tn*917ΩHU7*	Sandman et al., 1987
SB201 (MF1029)	*spoIIEΩspoIIE-gfp kan*	Garti-Levi et al., 2008
MF248	*amyE::P_*spoIID*_-gfp spc*	Fujita and Losick, 2003
PE128 (MF1027)	*amyE::P_*spoIIQ*_-gfp spc*	Eichenberger et al., 2001
CK55	*spoIIIEΩspoIIE-yfp cm*	Kaimer et al., 2009
PL412	*spoIIIE*::*spc*	Pogliano et al., 1997
CK153	*sftA*::*tet spoIIIE*::*spc*	Kaimer et al., 2009
PG26	*lacO cassette at 359°, thr::lacI-cfp*	Mascarenhas et al., 2002
AT62	*lacO cassette at 181°, thr::lacI-gfp*	Teleman et al., 1998
RL2242	*spo0A::spec*	Fawcett et al., 2000
PG28	*spo0H::spec*	Kind gift from P. Graumann's lab
PG37 (RL113)	*spo0H::cat*	Kind gift from P. Graumann's lab
1057	*trpC2 amyE*::*P_xyl_-ftsZ-cfp spc*	Feucht and Lewis, 2001
ATCC 9372™	*Bacillus atrophaeus*	American Type Culture Collection (ATCC)
ATCC 14884™	*Bacillus pumilus*	ATCC
DSM28	*Bacillus sphaericus*	German Collection of Microorganisms and Cell Cultures GmbH (DSMZ)

### Heat kill assay

Samples were collected in regular time intervals and serially diluted with sterile double distilled water (ddH_2_O). The number of spores was measured as heat-resistant (20 min at 80°C) colony-forming units (CFU) on LB plates. In parallel, viable cells were measured as total colony-forming units (CFU) on LB plates. In this case, serial dilutions were performed in sterile 0.9% NaCl. % sporulation = [spores/ml)/(cells/ml)] × 100% (Grossman and Losick, 1988). However, because the numbers of live vegetative cells decline after the start of the stationary phase, the percentage of sporulation during stationary phase was calculated relative to the number of viable cells reaching the stationary phase.

### Image acquisition

Cells were mounted on agarose gel pads containing S7_50_ medium (Harwood and Cutting, 1990) on microscope slides. Fluorescence microscopy was performed on a Zeiss Axio Imager A1 or a Zeiss Axio Observer Z1 microscope equipped with a CoolSnap HQ CCD camera (Photometrics) or pco.edge camera (scientific CMOS camera) respectively. The electronic data processing was conducted with *Metamorph* 6.3-Software (Meta Imaging Software) or VisiView Test-Version 2.1.1 (Visitron Systems GmbH). Fluorescence intensities were measured using the ROI manager tools of ImageJ (v1.48; Rasband,W.S., ImageJ, U.S. National Institutes of Health, Bethesda, Maryland, USA, http://imagej.nih.gov/ij/, 1997-2014) (Schneider et al., 2012).

## Results

### *B. subtilis* wild-type cells generate small round cells containing DNA during prolonged growth in minimal S7_50_ medium

Endospore formation is the preferred response of *B. subtilis* cells to different nutrient-limited conditions in minimal medium (Chubukov and Sauer, 2014). However, the extent to which this bacterium can adjust its structure and physiology during extended stationary phase in optimum growth condition is less known.

**Figure 1:**
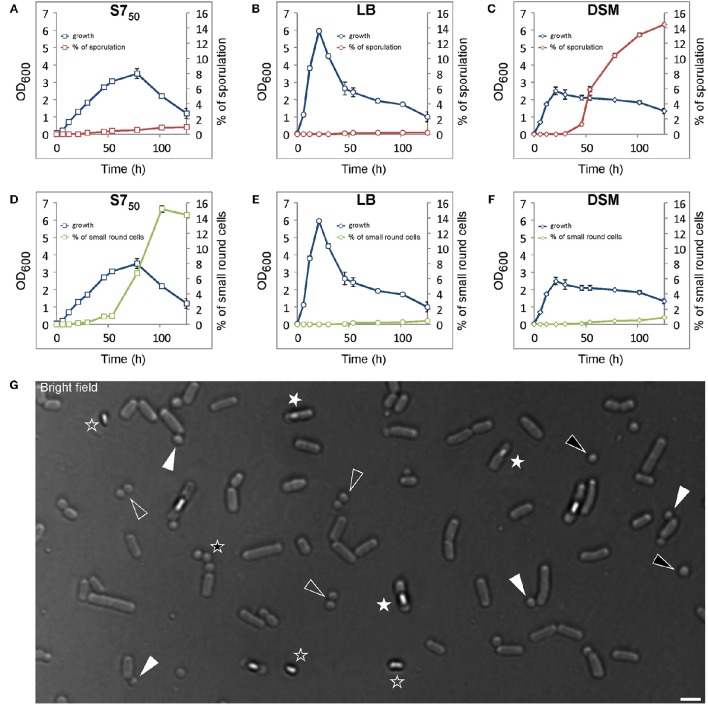
**Wild-type ***B. subtilis*** cells generate small round cells during prolonged growth**. Comparative analysis of growth and spore formation **(A–C)** and comparative analysis of growth and small round cells formation **(D–F)** in different media over time. Error bars represent standard deviations of the means of two independent experiments. Small round cells counts and sporulation data are shown in Supplemental Data (Table S1–S4). **(G)** Representative view of cells acquired at time point 100 h grown in S7_50_ at room temperature. White triangles indicate small round cells still attached to their larger siblings, black triangles indicate free small round cells, white asterisks indicate sporulated cells, black asterisk or empty triangles indicate a triplet or doublets of small round cells respectively from consecutive polar divisions. Empty asterisks indicate released spores. Scale bars 2 μm.

To follow sporulation behavior of wild-type *B. subtilis* cells during prolonged stationary growth under laboratory conditions, minimal S7_50_ medium (Grossman and Losick, 1988; Harwood and Cutting, 1990) was chosen for this study, which is frequently used to grow *B. subtilis*. For comparative analysis Lysogeny Broth (LB) (Bertani, 2004) and sporulation medium DSM (Schaeffer et al., 1965b) were also used. Cells were grown at room temperature (25°C) with shaking at 200 rpm and samples were collected at regular time intervals for OD_600_ measurement, microscopy analysis and evaluation of the % of sporulation. Whenever *B. subtilis* was grown in S7_50_ minimal medium, LB broth or DSM medium, sporulation occurred earlier in S7_50_ and DSM media (~ 12 h) compared to LB broth (~ 21 h). In all media, the percentage of spores in relation to the total viable population increased with time and yielded 0.94 and 0.21% in S7_50_ medium and LB broth, respectively, after 126 h (Figures 1A,B; the sporulation data are shown in Tables S1,S2). Meanwhile, spores readily accumulated in DSM medium and reached 14.5% of total viable counts after 126 h (Figure 1C; the sporulation data are shown in Table S3). Spores were formed already during exponential growth phase in S7_50_ medium (Figure 1A), in contrast to DSM medium and LB broth, in which spores appeared only when cell density declined (Figures 1B,C). These observations indicate that spore development in DSM medium and LB broth arose upon nutrient exhaustion, while in S7_50_ medium the heterogeneity of the cell population with respect to sporogenesis started earlier during growth.

Interestingly, in addition to spores, the formation of small round cells was observed that appeared in a way similar to the well-characterized minicells (Adler et al., 1967; Reeve et al., 1973) (Figures 1D–G). Appearance of this cell type could be detected in all media, but was most prominent in S7_50_, in which a rapid increase was noted at the transition between logarithmic growth and stationary phase. The small round cells eventually constituted 15.2% of the total cell counts (1750 cells counted; small round cells counts data are shown in Table S4) in S7_50_ (Figure 1D) compared to 0.45 or 0.9% in LB or DSM, respectively (Figures 1E,F, the small round cells counts data are shown in Tables S5,S6). The small round cells exhibited diameters varying from 0.7 to 1.15 μm (150 cells measured) (Figure 1G). The undomesticated *B. subtilis* strain (NCIB 3610) as well as other species such as *Bacillus atrophaeus, pumilus*, and *sphaericus* (Figure S1) also produced small round cells, suggesting that this phenotype is not a trait of the lab strain PY79 and may be conserved among endospore-forming bacteria.

### Small round cells evolve from the abortion of sporulation

**Figure 2:**
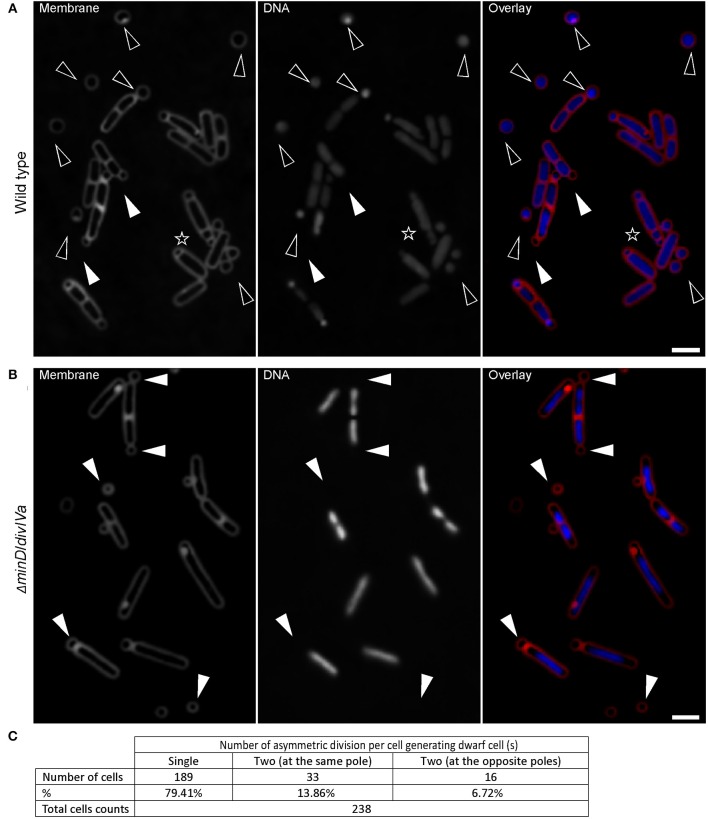
**Wild-type ***B. subtilis*** cells generate small round cells containing DNA. (A)** Wild-type cells showing that almost all small round cells contain DNA (black triangles). White triangles indicate two small round cells devoid of DNA. 98.33 ± 0.58% (mean ± *SD* of three independent experiments; > 250 cells counted each) of small round cells generated from wild-type cells contain DNA, while minicells generated from a *B. subtilis minD*/*divIV* double mutant **(B)** are all devoid of DNA. White triangles point out some minicells. The black asterisk in panel **(A)** indicates a cell with multiple attempts of sporulation. Cells were grown in S7_50_ for about 100 and 28 h for wild-type and *minD*/*divIV* double mutant cells respectively. **(C)** Quantification of the frequency of the number of asymmetric divisions occurring per cell generating dwarf cell (s). Scale bars 2 μm.

A brief analysis of the small round cells showed that they were significantly different from minicells because of the presence of DNA in these cells visualized by DAPI staining (Figure 2A), which was absent in minicells from a mutant defective in proper placement of the cell division machinery (Figure 2B).

The question that immediately arose was to find out the origin of these small round cells. The accumulation of small round cells in the growth medium as the culture reached the stationary phase where sporulation events should have happened more frequently suggests that the occurrence of small round cells in S7_50_ could be the result of failed spore development.

**Figure 3:**
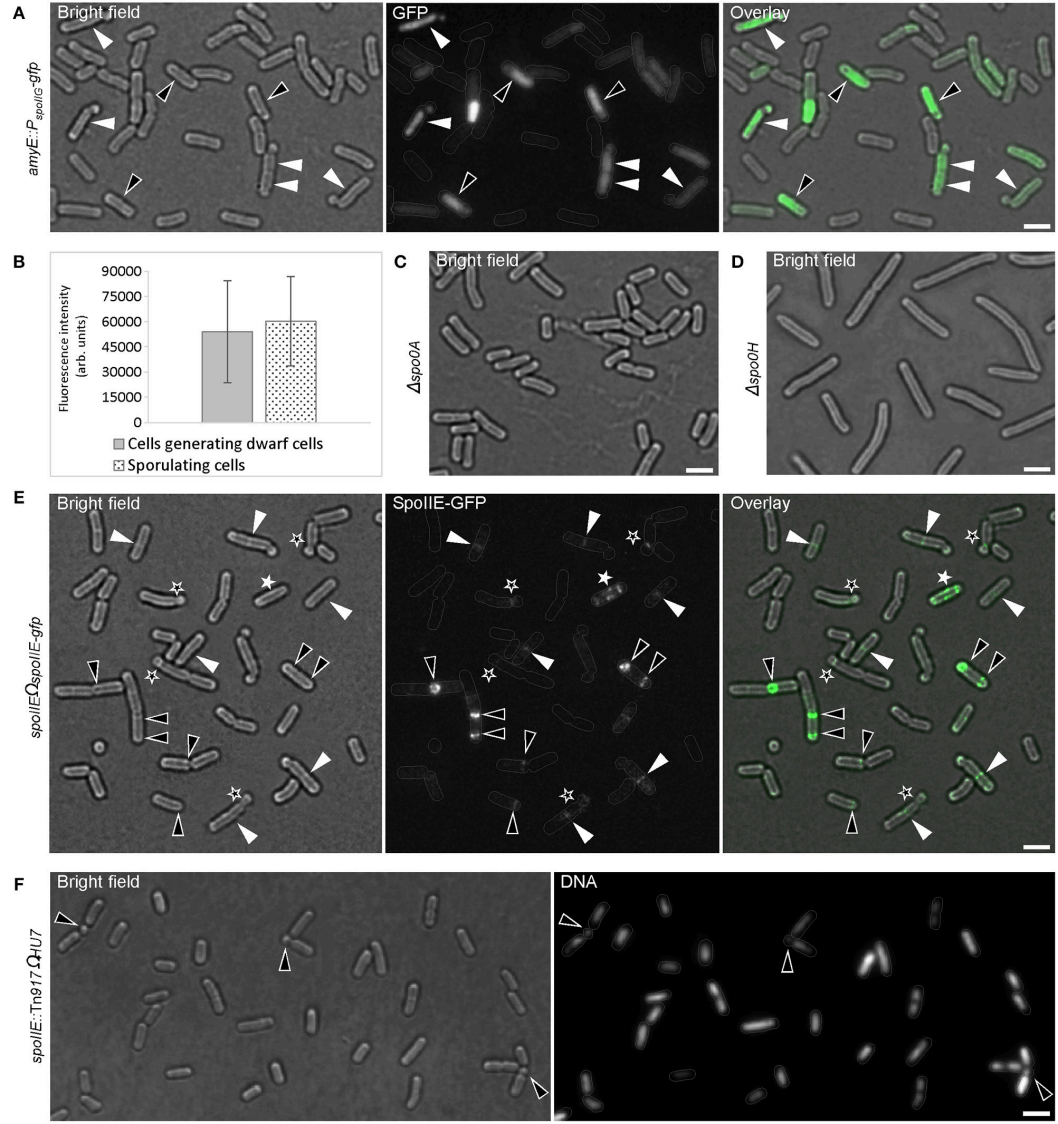
**Early sporulation genes (onset of sporulation) are active during formation of small round cells. (A)** Cells expressing a fusion of *gfp* to the Spo0A-controlled *spoIIG* gene to visualize the activity of Spo0A. Black and white triangles indicate sporulating cells and cells generating small round cells respectively. **(B)** Analysis of GFP intensity in cells shown in panel **(A)**. The difference in the GFP intensity of sporulating cells compared with cells generating small round cells was evaluated by the Mann-Whitney test and a *p*-value of > 0.05 (0.1765) was considered statically non-significant. 178 and 193 cells were measured for cells generating small round cells and sporulating cells respectively. Error bars represent standard deviations of the means. Fluorescence intensities are in arbitrary units (arb. units). Error bars represent standard deviations of the means. *spo0A* **(C)** or *spo0H* **(D)** mutants are unable to generate small round cells. **(E)** Cells expressing SpoIIE fused to GFP. Black triangles indicate polar E-rings. White asterisks indicate the helical distribution of SpoIIE-GFP. White triangles indicate the presence of E-rings at mid-cell position. Black asterisks indicate E-rings at the septum of the small round cells. **(F)** *spoIIE* mutant cells also generate small round cells (black triangles), but less frequently than the wild-type strain. Gray lines show the contour of the cells. Scale bars 2 μm.

*B. subtilis* has different developmental states that are mostly controlled by Spo0A, the master regulator of the stationary phase. To further confirm that there is a link between sporogenesis and small round cells formation, the expression level of Spo0A using a *gfp* fusion to the *spoIIG* promoter was monitored. *spoIIG* is a sporulation gene which is controlled by Spo0A (Satola et al., 1992). Spo0A is the sporulation initiator protein and its intracellular level determines the entry of cells into the sporulation process. High levels of Spo0A are required for the activation of sporulation-specific genes (Fujita et al., 2005). Figures 3A,B show that the intensity of GFP (expressed from *spoIIG* promoter from an ectopic locus) fluorescence signals in cells producing small round cells (54171.84 ± 30261.937 arb. Units; mean ± *SD* of 178 measurements) was similar to that of sporulating cells (60386.68 ± 26532.14 arb. Units; mean ± *SD* of 193 measurements). This suggests that the Spo0A concentration in cells producing the small round cells was sufficient to trigger sporulation. Moreover, all cells producing the small round cells exhibited GFP fluorescence suggesting that small round cells formation requires the activity of Spo0A. Indeed, I also investigated a *spo0A* mutant which is known to arrest sporulation at stage 0 (Piggot and Coote, 1976). No single small round cells (observation from three independent experiments, 300 cells analyzed each) could be detected in this strain (Figure 3C). Cells lacking *spo0H* gene, which codes for σ^H^ that directs genes expression at an early stage of sporulation, also did not generate small round cells (observation from two independent experiments, 350 cells analyzed each). However, for unknown reasons *spo0H* deletion strains investigated in this work exhibited elongated shaped cells (Figure 3D). Because sporulation is the unique developmental state requiring an asymmetric septation, these findings are consistent with the idea that the small round cells can only be formed upon initiation of spore development. Thus, the small round cells evolve from the inability of cells to complete sporulation. Of the cells generating the small round cells (the small round cells still being attached to their larger siblings), 20% (Figure 2C, 238 cells counted) underwent more than one asymmetric division at the same pole or opposite poles (Figures 1G, 2A, black asterisk), indicating that a failure to develop a spore does not imply that the cell returns to the vegetative state, and thereby allowing a subsequent reinitiation of sporulation. Thus, the initial sporulation signals are rather strong and remain stable in cells producing the small round cells.

### *B. subtilis* mutant defective in proper polar septation forms fewer small round cells

Asymmetric cell division in *B. subtilis* is the second major morphological change observed during sporulation (Schaeffer et al., 1965a). My observations indicated that the development of the spore septum must be required for the small round cells formation. To test whether the ability to form proper polar septa affects the occurrence of the small round cells, the bifunctional SpoIIE protein was examined. SpoIIE is involved in asymmetric division and is required for the establishment of compartment-specific activation of transcription factors (Barák et al., 1996). SpoIIE is proposed to play a role in the switch from one Z-ring observed during vegetative growth to two polar Z-rings when cells enter sporulation (Khvorova et al., 1998). Consequently, SpoIIE also assembles into rings (E-ring) that are dependent on Z-ring formation (Arigoni et al., 1995; Levin et al., 1997; King et al., 1999). Figure 3E shows rings and helical patterns of SpoIIE-GFP localization. 92% of small round cells (146 cells analyzed) had E-rings at their septum. The remaining small round cells showed no or very faint SpoIIE-GFP signals at their septum (Figure 3E). Interestingly, an E-ring also frequently formed at the mid cell position indicating that the interaction of SpoIIE with the vegetative Z-ring precedes the polar relocalization of the Z/E-rings via the helical pattern distribution of both proteins (Ben-Yehuda and Losick, 2002) (Figure 3E).

The precise role of SpoIIE in this dynamic relocalization of FtsZ is still unclear. However, in the absence of SpoIIE, sporulating cells are still able to assemble polar Z-rings and to form asymmetrically positioned septa (Levin et al., 1997; Wu et al., 1998). These septa are rather thick and similar to those formed during vegetative growth. The literature suggests that SpoIIE may regulate cell wall synthesis at the asymmetric division sites (Piggot and Coote, 1976; Illing and Errington, 1991; Barák and Youngman, 1996) and may play a role in peptidoglycan remodeling during engulfment (Campo et al., 2008). Indeed, a recent study indicated that SpoIIE recruits the morphogenic protein RodZ at the asymmetric septum during sporulation where both proteins might be involved in peptidoglycan thinning during engulfment (Muchova et al., 2016). The second distinct function of SpoIIE during sporulation is the activation of σ^F^ in the forespore due to its phosphatase activity (Duncan et al., 1995; Arigoni et al., 1996; Feucht et al., 1996). Therefore, a *spoIIE* mutant arrests spore development at stage II (Margolis et al., 1991). The *spoIIE* null mutant analyzed in this work also generated the small round cells (Figure 3F), but only up to 3.0 ± 0.6% (mean ± *SD* of three independent experiments; 247 cells counted each); a frequency that is 5-fold less than that observed in wild-type strain. This finding is consistent with the previous report that *spoIIE* null mutants have a noticeably reduced frequency of polar septum formation (Barák and Youngman, 1996) and shows a correlation between the ability to form polar septa and the generation of small round cells. Taken together, these findings support the idea that the small round cells formation requires the asymmetric septa involved in sporulation.

### σ^E^ or σ^F^ are not active during formation of small round cells

**Figure 4:**
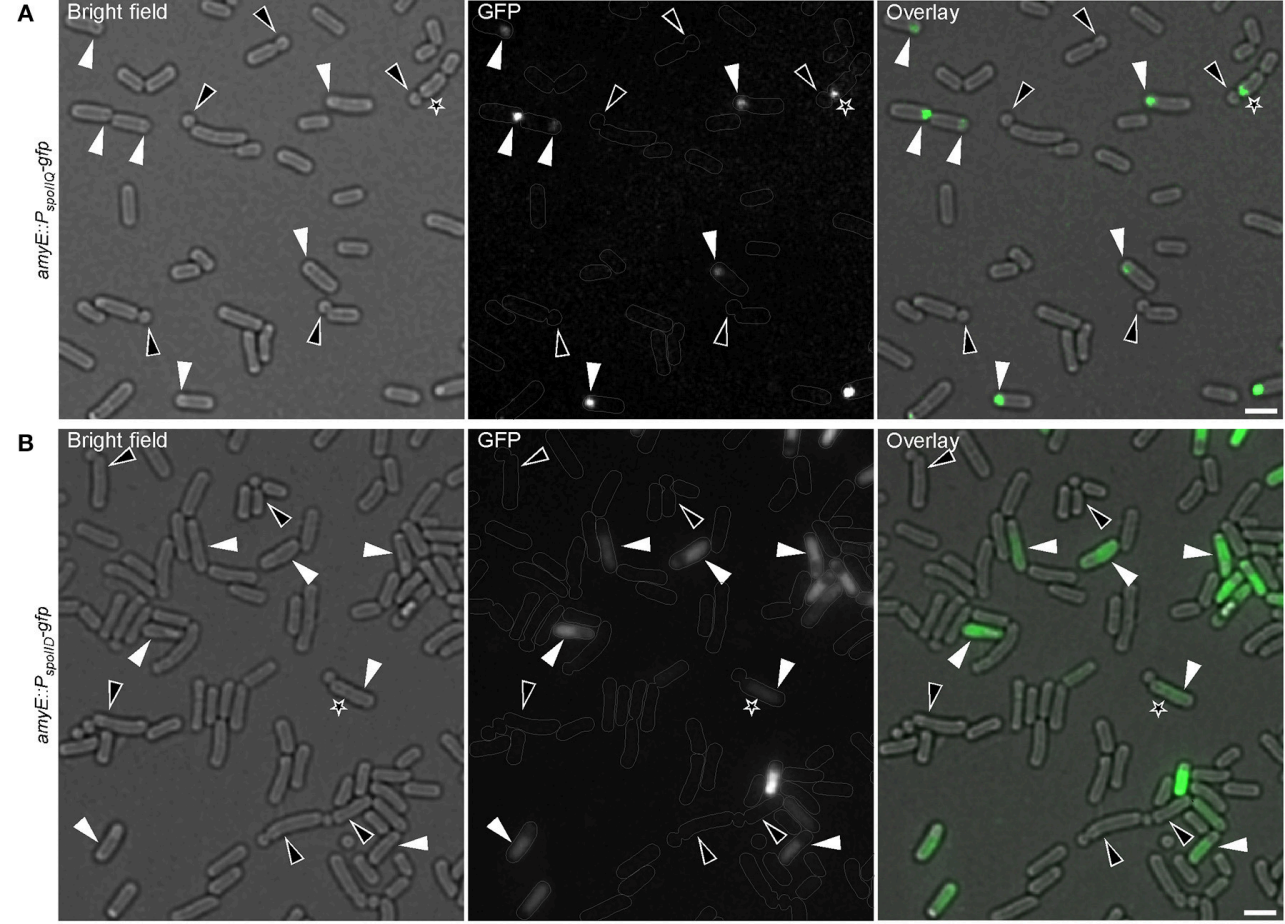
**Compartmentalized activation of sporulation-specific sigma factors is not established during formation of small round cells**. Cells expressing a fusion of *gfp* to the σ^F^-controlled *spoIIQ* **(A)** or to σ^E^-controlled *spoIID* **(B)** genes were applied to visualize the activity of σ^F^ or σ^E^, respectively. White triangles indicate sporulating cells with active σ^F^ or σ^E^. Black triangles indicate cells dividing asymmetrically with no apparent activity of any sigma factor. Black asterisks indicate cells that had abortive sporulation followed by a successful attempt as judged by the attached small round cells. Gray lines show the contour of the cells. Scale bars 2 μm.

Because E-ring signals could be also visualized at the small round cells septa (Figure 3E), I investigated whether σ^E^ or σ^F^ were indeed expressed during the formation of small round cells in the former mother cell or forespore respectively. The activity of σ^E^ or σ^F^ was visualized using strains bearing *gfp* fusions to *spoIID* or *spoIIQ* promoter of whose expression is controlled by σ^E^ or σ^F^, respectively (Rong et al., 1986; Londoño-Vallejo et al., 1997). Figure 4 shows that σ^E^ and σ^F^ were active in cells undergoing sporulation, but not in those generating small round cells. Sporulating cells showed discrete curved septa indicating the engulfment of the forespore at the cell pole while a true polar cell division was often marked by a clear invagination of the cell wall (Figures 4A,B). In some cases, cells turned on σ^E^ or σ^F^ following the formation of the small round cell (Figures 4A,B, black asterisks). This observation is consistent with the above finding that cells generating the small round cells retain sporulation signals and can attempt another round of sporulation initiation. Unexpectedly, the forespores were always formed at poles where small round cells had been generated, suggesting the presence of specific markers at the cell poles. These markers would eventually drive a subsequent sporulation event at the same pole. Thus, the formation of the small round cells does not require the activities of σ^E^ or σ^F^ but σ^H^ controlling the early stationary phase genes involved in the initiation of sporulation.

### Chromosomal DNA is actively translocated into the small round cells

At the onset of sporulation, as cells possess two chromosomes, the origins of replication are targeted to the opposite cell poles by RacA in a DivIVA-dependent manner (Ben-Yehuda et al., 2003). This mechanism ensures that the future sporangium gets a copy of the chromosome at both poles. When the asymmetric division is taking place, SpoIIIE, a DNA translocase assembles at the closing septum and actively pumps the remaining chromosome from the mother cell into the forespore (Wu et al., 1995; Wu and Errington, 1997; Bath et al., 2000).

**Figure 5:**
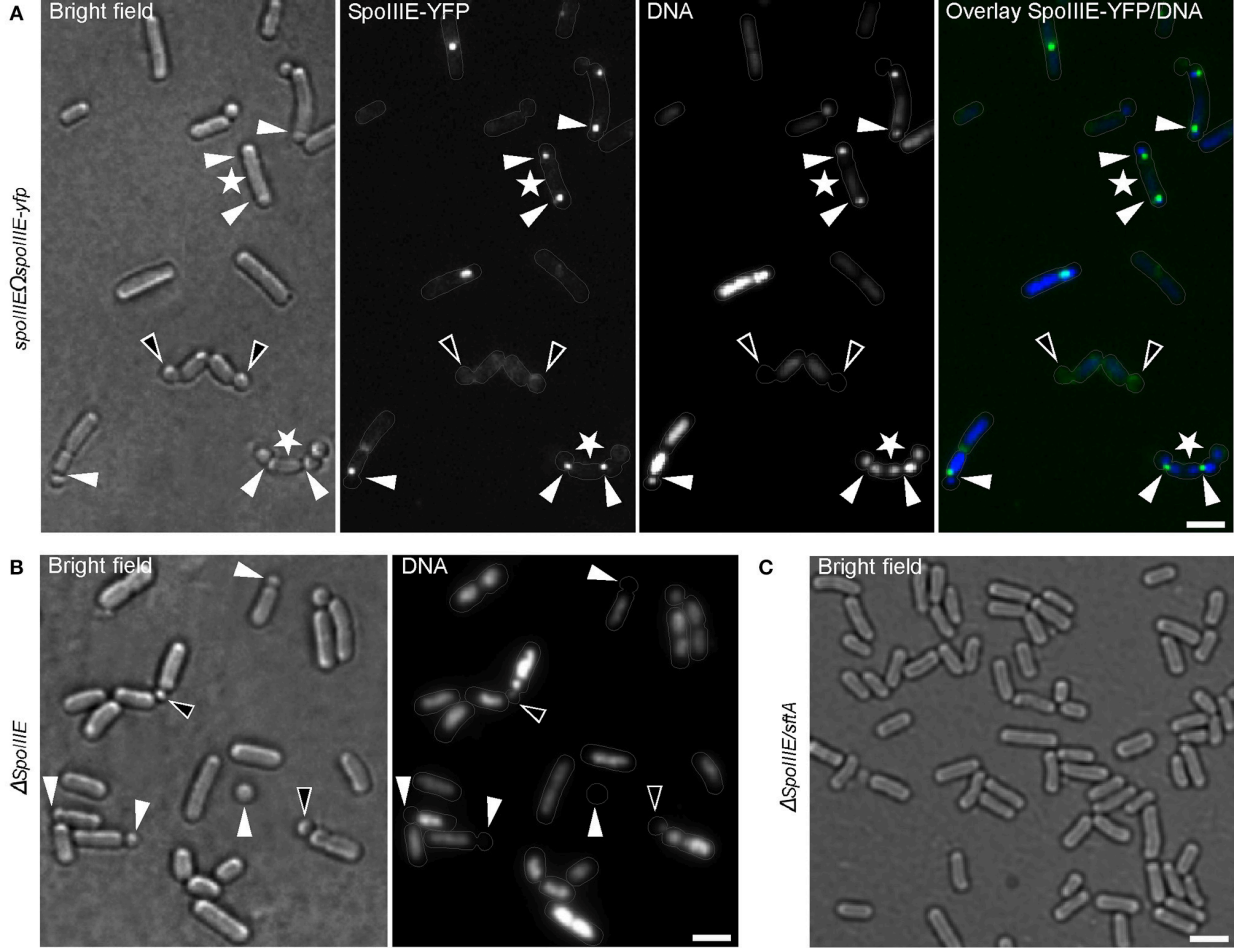
**Chromosomal DNA is actively translocated into the small round cells. (A)** *B. subtilis* cells expressing DNA translocase SpoIIIE fused to YFP. White triangles indicate SpoIIIE-YFP focus at the asymmetric septum. White asterisks indicate few cases where bipolar asymmetric septa persist and generate two small round cells from a single cell. Black triangles indicate small round cells without DNA lacking SpoIIIE-YFP foci at their septa. **(B)** *spoIIIE* mutant cells showing an increased number of small round cells without DNA (white triangles). Black triangles indicate small round cells apparently containing only residual amounts of DNA. **(C)** Cells bearing null mutations in both *spoIIIE* and *sftA* are unable to generate small round cells. Gray lines show the contour of the cells. Scale bars 2 μm.

This raises the question whether DNA is actively transported into the small round cells or simply pinched-off from the bulk nucleoid by the closing septum. To explore this question, I examined a strain expressing SpoIIIE fused to YFP as a sole source of protein during the formation of small round cells. SpoIIIE-YFP accumulated at the asymmetric septum, as previously reported (Wu et al., 1995; Wu and Errington, 1997; Bath et al., 2000) (Figure 5A). Interestingly, a SpoIIIE-YFP foci was also present at the asymmetric septum of most of the small round cells containing DNA (90% of 213 cells counted), but absent at the septum of all empty small round cells (empty small round cells represent about 1.67% of the small round cells) (Figure 5A). Thus, similar to the sporulation process, SpoIIIE is also required for DNA translocation during the formation of small round cells. Surprisingly, up to 60% (180 cells counted) of small round cells produced by a *spoIIIE* null strain contained DNA (Figure 5B). This observation suggests that another cellular factor is implicated in this process. Such factor could be the *B. subtilis* DNA translocase SftA (septum-associated FtsK-like translocase of DNA). SftA belongs to the SpoIIIE/FtsK-like protein family found in *B. subtilis*, but lacks the transmembrane domain (Biller and Burkholder, 2009; Kaimer et al., 2009). It has been proposed that SftA acts first at the cell division site by clearing the sister chromosomes and that SpoIIIE only comes into play when chromosomes get trapped at the closing septum (Biller and Burkholder, 2009; Kaimer et al., 2009; Wu, 2009). However, recent investigations using super-resolution microscopy revealed that SpoIIIE is consistently present at the invaginating septum (Fiche et al., 2013), suggesting that both proteins are synchronously recruited to the division machinery. A *spoIIIE*/*sftA* double null mutant neither produced small round cells nor sporulated (Figure 5C), a finding consistent with the correlation between spore and the small round cells formation. Thus, similar to their respective function during vegetative growth, SpoIIIE, and SftA act synergistically to ensure proper chromosome partitioning/translocation during sporulation or subsequent small round cells formation when the sporulation process fails.

### Small round cells contain a full chromosome

**Figure 6:**
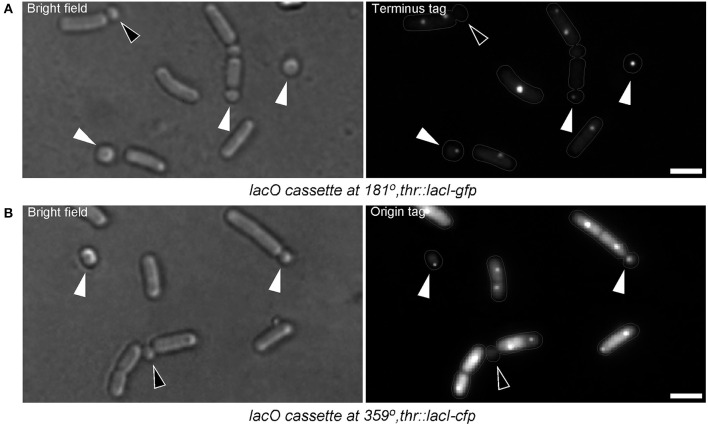
**Small round cells contain full chromosomes. (A)** *B. subtilis* cells harboring a *lacO* cassette at 181° (terminus region of the chromosome). White triangles indicate small round cells with terminus region signal, which is a proof that the cells possess the full chromosome. The black triangle indicates a small round cell which has not completed DNA translocation (both terminus regions are still in the larger cell compartment). **(B)** *B. subtilis* cells harboring a *lacO* cassette at 359° (origin region of the chromosome). Nearly all small round cells contain the origin region (white triangles). Black triangle indicates a small round cell lacking origin signal. Terminus region tagged cells were grown in S7_50_. For unknown reasons, origin region tagged cells hardly sporulated or generated small round cells in S7_50_. However, when grown in S7_50_ diluted 1:1 with LB, sporulation and small round cells formation could be observed (but less frequently compared with the wild-type cells grown in S7_50_). Somehow, in this medium, cells exhibited high level of diffused fluorescence probably due to the higher expression of GFP-LacI. Gray lines show the contour of the cells. Scale bars 2 μm.

It was interesting to find out if the translocation of DNA into the emerging small round cells might be affected while abortion of the sporulation occurs. In other words, do the small round cells contain full chromosomes? Assuming that 2/3 of the forespore chromosome, which remains in the mother cell (Wu and Errington, 1994), is translocated in an ordered manner (i.e., the terminus of the chromosome being translocated last), this question was examined by visualizing the presence of the terminus (*terC*) region of the chromosome in the small round cells. Figure 6A shows that about 50% (103 cells counted) of the small round cells contained the *terC* region while 98.33% of the small round cells contained DNA (Figure 2A). The discrepancy between the number of cells containing *terC* and the total number of cells containing DNA could be due to incomplete translocation of the chromosome into the forespore. In agreement with this, about 99% (68 cells counted) of the small round cells were found to contain *oriC* (Figure 6B). Therefore, the ~2% of anucleate small round cells may suggest a release of the *oriC* region from the forespore pole and subsequent retraction of the chromosome into the mother cell compartment. This could happen when opposing signals to commitment are sensed earlier in the developmental process. However, although there is no evidence that *B. subtilis* can reverse commitment to sporulation by retracting the chromosome, it should be noted that the chromosome will be retracted if the origin is not in the forespore (Becker and Pogliano, 2007). In any case, the high probability that the small round cells inherit a full copy of the chromosomal DNA increases the chances of viability for these cells.

### Small round cells resume growth upon addition of fresh growth medium

**Figure 7:**
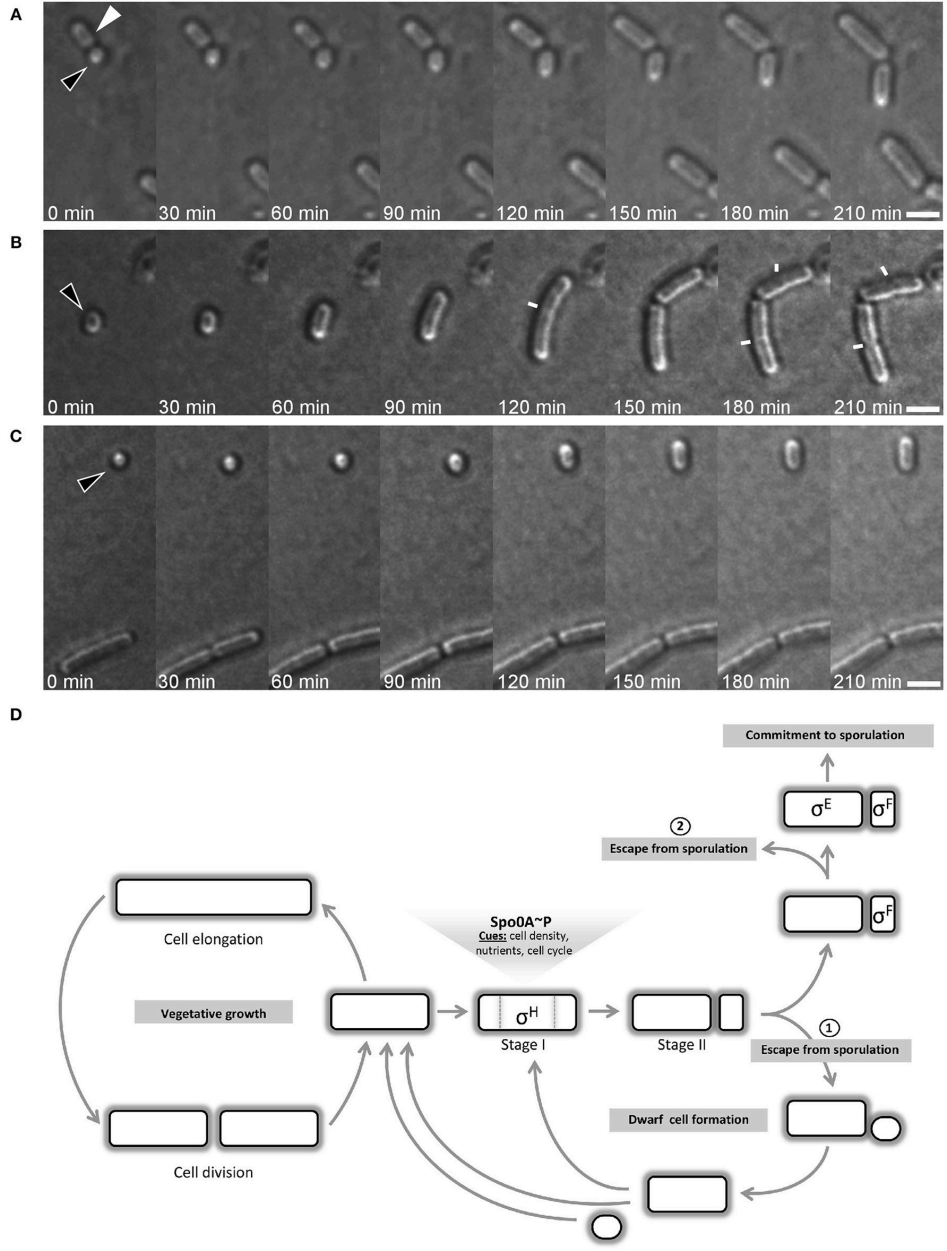
**Time-lapse microscopy of growing small round cells**. Black triangles indicate small round cells that grow back to rod shape and white triangle indicate a former mother cell resuming growth. Images were acquired every 30 min. Small round cells are of different size at the start of the time-lapse: “medium” **(A)**, “large” **(B)**, and “tiny” **(C)**. In **(B)**, white lines indicate the septum of dividing cells. Scale bars 2 μm. **(D)** *B. subtilis* cell cycle depicting the bifurcation in the spore development process. After the initiation phase of sporulation and the establishment of the asymmetric septum, the cell can escape from sporulation by generating a small round cell, which I name “dwarf” cell as it is generated by wild-type cells. However, the sporulation signals can remain stable in the former mother cell and a second round of the process is often initiated. Dwarf cells and former mother cells are able to return to rod shape and resume vegetative growth. The commitment to sporulate requires that both sigma factors are activated. The activation of sigma factors is not simultaneous and the activation of σ^F^ occurs first. Before σ^E^ is activated, cell can still abort the sporulation process, as reported by Narula et al. (2012).

Sporulating cells lacking σ^F^ are unable to complete spore development and end up with an empty mid-cell compartment flanked by two opposite forspores containing each a full copy of the chromosome. These forespores are able to resume vegetative growth when shifted into rich medium (Dworkin and Losick, 2005). A key question of this work was whether the small round cells were dead-end structures or whether they could resume normal growth upon addition of fresh medium. To test this, I monitored the growth of small round cells by time-lapse microscopy. Cultures of *B. subtilis* generating small round cells were diluted into fresh growth media and grown for 60 min at room temperature before time-lapse imaging. Strikingly, the small round cells were able to return to the rod shaped form (Figures 7A–C, Figure S2, and Movie S1). Different sizes of small round cells were monitored for growth. Out of 49 cells analyzed, 47 cells extended into rods. The small round cells observed did not extend at the same speed. The speed of growth was dependent on the size of the small round cells at the beginning of the experiment. The larger the diameter size of small round cells, the faster they reached their first division. Among the 47 cells that extended into rods, 16 cells that had diameter sizes ranging from 0.95 to 1.15 μm cells divided after 2.5–3 h and 21 cells that had diameter sizes ranging from 0.8 to 0.9 divided after 3.5–4 h. The rest of the cells (10 cells) with smaller diameter sizes (~0.7 μm) did not did divide during the time course of the time-lapse experiment, which was ~4 h.

Taken together, these experiments suggest that the here-described small round cells could provide an escape route for cells that are unable to continue the sporulation process.

## Discussion

Although spore development is generally assumed to be a well-studied process, sporulation has not yet been stringently assessed in conditions that are not optimized to induce sporulation. In this work, I have monitored the sporulation behavior of wild-type *B. subtilis* in standard minimal S7_50_ medium during extended stationary-phase growth. For comparative analysis, I have used the sporulation DSM and the nutritionally rich LB media. In DSM, as expected, the major response of *B. subtilis* cells to stationary-phase growth was the formation of spores due to nutrient limitation (Schaeffer et al., 1965b).

In LB, *B. subtilis* cells yielded poor sporulation efficiency. What then can cause inhibition of sporulation in LB? It was shown that in contrary to the conventional thinking, LB medium is a rather carbon-source limited (Sezonov et al., 2007). Tryptone and yeast extract which are the main components of LB are mostly composed of oligopeptides (Bertani, 1951). The catabolizable amino acids recovered from these oligopeptides represent the main carbon source. Their degradation during growth results in excretion of the excess ammonium, which alkalinizes the growth medium. Thus, the inhibition of sporulation observed in LB is neither due to catabolite repression nor the acidification of the culture medium and therefore, remains enigmatic.

The major finding comes from the growth in minimal medium. One could expect that the sporulation would readily occur, but this was not the case. I show that instead of spores, cells frequently generate small round shaped offsprings as the culture reaches the stationary phase. The small round cells are morphologically similar to spherical cell morphology generated by *Escherichia coli* during stationary-phase growth. However, in contrast to small round cells that are generated by asymmetric septation, small spherical *E. coli* cells arise from mid cell division of cells that have decreased their size and changed their shape in response to starvation or stationary-phase conditions (Lange and Hengge-Aronis, 1991).

The small round cells are also morphologically similar to minicells. Minicells are mostly described as originating from cells lacking the MinCD components of the cell division machinery or mutants unable to properly segregate their chromosome (Adler et al., 1967; Reeve et al., 1973; de Boer et al., 1989; Levin et al., 1992; Varley and Stewart, 1992; Wu and Errington, 2004; Bernhardt and de Boer, 2005). An *mreBH* (*mreB* paralog in *B. subtilis*) mutant has been described to generate minicells containing DNA (Soufo and Graumann, 2010) similar to *spoVG* mutant cells during sporulation (Matsuno and Sonenshein, 1999). Recently, *B. subtilis* cells expressing the cytosolic part of RodZ was shown to generate comparable type of minicells (Muchova et al., 2013). However, the small round cells observed here originate from wild-type cells and no mutations were employed. This implies that the small round cells examined in this work are unprecedented. Therefore, to distinguish them from the well-known minicells, I propose to name them “dwarf cells.” The observation of dwarf cells in several different, spore-forming bacteria suggests that their appearance is not limited to *B. subtilis*, but might be general to spore formers.

This work clearly shows that cells generating dwarf cells are physiologically similar to cells initiating sporulation. Indeed, both types of cell expressed the same level of Spo0A. I could show that cells generating dwarf cells have well initiated sporulation, but for yet unknown reasons, the process aborts and dwarf cells are produced. Therefore, it seems that dwarf cells evolve from former forespores. The spore developmental process seems to be perturbed after the asymmetric division has taken place. Interestingly, cells can undergo one or two re-initiations as judged by multiple dwarf cell formations showing that the initial decision to sporulate persists despite failure.

The presence of SpoIIE—which is thought to regulate the thickness of the septum during sporulation (Piggot and Coote, 1976; Illing and Errington, 1991; Barák and Youngman, 1996)—at the septum of the dwarf cells raises the question whether their septum could be thin like the sporulation septum or thick like the septum of vegetative cells. Because dwarf cells separate from the former mother cells, it seems likely that their septum is similar to that of the vegetative cells. In this case, SpoIIE would not be needed for the synthesis of the septum of the dwarf cells. Indeed, the data show that some dwarf cells do not harbor SpoIIE-GFP signal at their septum. This observation suggests that SpoIIE might be removed from the septum of the dwarf cells either by the degradation of the protein or by the disassembly of the E-ring and its reassembly at the medial position in the former mother cell in case of re-initiation of sporulation. In any case, it will be interesting to characterize the exact nature of the septum built during the formation of dwarf cells.

Narula and collaborators previously reported abortion events during sporulation. They noticed that, until the σ^E^ is activated in the mother cell, sporulating cells can fail to engulf the forespore and either become dormant or resume vegetative growth (Narula et al., 2012). However, neither dwarf cell formation nor multiple initiations of sporulation events were reported. My experiments suggest that cells can escape from sporogenesis very early in the process. Frequently, cells are unable to activate σ^E^ and σ^F^ after polar division and rather generate dwarf cells, which can re-enter normal growth as rod shaped cells (Figure 7D). The inability of cells lacking Spo0A (the master regulator of sporulation initiation) or σ^H^ to generate dwarf cells, the detection of Spo0A activity in all cells generating dwarf cells and the ability of dwarf cells to grow back to rod shape cells strongly suggest that dwarf cell formation is a new escape route from sporulation. Thus, when cells cannot further proceed with the sporulation process after the polar septation, a “true” asymmetric cell division appears to be a proper rescue mechanism for the pre-divisional sporangium.

This cellular event, which is described here for the first time, adds a further layer of complexity to the complex bacterial developmental pathways. Further investigations are needed to characterize environmental and physiological cues that cause the early abortion of sporulation (i.e., before the expression of sigma factors) described in this work. On the other hand, dwarf cells could be an interesting tool for the investigation of cellular components involved in the determination of rod shape in bacteria.

## Author contributions

HJDS conceived, designed and performed the experiments, analyzed the data, and wrote the paper.

### Conflict of interest statement

The author declares that the research was conducted in the absence of any commercial or financial relationships that could be construed as a potential conflict of interest.
