# Supplementary material for: A Novel Cell Type Enables B. subtilis to Escape from Unsuccessful Sporulation in Minimal Medium
Source: Front Microbiol. 2016 Nov 11;7:1810. doi: 10.3389/fmicb.2016.01810 (PMC5104909; doi:10.3389/fmicb.2016.01810)
Supplement: Supplementary file 2 [file DataSheet1.PDF]

Supplemental Data

Table S1. Sporulation data in $S7_{50}$

	Optical density (OD)		Mean of OD	Standard deviation (SD) of OD	CFU/ml of vegetative cells		CFU/ml of spores		% of sporulation		Mean % of sporulation	SD % of sporulation
Time (h)	Exp. #1	Exp. #2			Exp. #1	Exp. #2	Exp. #1	Exp. #2	Exp. #1	Exp. #2		
0	0.05	0.05	0.05	0	NA	NA	0	0	0	0	0	0
6	0.2	0.21	0.205	0.00707107	36000000	38000000	0	0	0	0	0	0
12	0.68	0.72	0.7	0.02828427	128000000	131000000	200	210	0.00015625	0.00016031	0.00015828	2.0277E-06
21	1.25	1.33	1.29	0.05656854	239000000	244000000	778	782	0.00032552	0.00032049	0.00032301	2.5156E-06
30	1.79	1.84	1.815	0.03535534	318000000	323000000	540000	600000	0.16981132	0.18575851	0.17778492	0.0079736
46	2.69	2.72	2.705	0.0212132	498000000	506000000	1550000	1600000	0.31124498	0.31620553	0.31372526	0.00248028
54	3.01	3.1	3.055	0.06363961	556000000	560000000	2360000	2600000	0.42446043	0.46428571	0.44437307	0.01991264
78	3.3	3.7	3.5	0.28284271	676000000	682000000	4010000	3600000	0,59319527	0,52785924	0,5605273	0,032668
102	2.1	2.3	2.2	0.14142136	387000000	389000000	5800000	6000000	0,85798817	0,8797654	0,8688768	0,0108886
126	1	1.4	1.2	0.28284271	261000000	264000000	6700000	6000000	0,99112426	0,8797654	0,9354448	0,0556794

Table S2. Sporulation data in LB

	Optical density (OD)		Mean OD	Standard deviation (SD) of OD	CFU/ml of vegetative cells		CFU/ml of spores		% of sporulation		Mean % of sporulation	SD % of sporulation
Time (h)	Exp. #1	Exp. #2			Exp. #1	Exp. #2	Exp. #1	Exp. #2	Exp. #1	Exp. #2		
0	0.05	0.05	0.05	0	NA	NA	0	0	0	0	0	0
6	1.15	1.1	1.125	0.03535534	115000000	113000000	0	0	0	0	0	0
12	3.9	3.72	3.81	0.12727922	387000000	365000000	0	0	0	0	0	0
21	6.02	5.9	5.96	0.08485281	593000000	577000000	58	53	9.7808E-06	9.1854E-06	9.4831E-06	2.9767E-07
30	4.43	4.6	4.515	0.12020815	452000000	438000000	3000	2770	0.0005059	0.00048007	0.00049299	1.2916E-05

46	2.9	2.38	2.64	0.36769553	281000000	273000000	700000	610000	0.11804384	0.10571924	0.11188154	0.0061623
54	2.6	2.24	2.42	0.25455844	263000000	251000000	1030000	980000	0.17369309	0.16984402	0.17176855	0.00192453
78	2	1.85	1.925	0.10606602	195000000	182000000	1120000	1090000	0.18887015	0.18890815	0.18888915	1.8997E-05
102	1.8	1.65	1.725	0.10606602	178000000	159000000	1230000	1210000	0.2074199	0.20970537	0.20856264	0.00114274
126	1.2	0.8	1	0.28284271	170000000	149000000	1250000	1240000	0.21079258	0.21490468	0.21284863	0.00205605

Table S3. Sporulation data in DSM

	Optical density (OD)		Mean of OD	Standard deviation (SD) of OD	CFU/ml of vegetative cells		CFU/ml of spores		% of sporulation		Mean % of sporulation	SD % of sporulation
Time (h)	Exp. #1	Exp. #2			Exp. #1	Exp. #2	Exp. #1	Exp. #2	Exp. #1	Exp. #2		
0	0.05	0.05	0.05	0	NA	NA	0	0	0	0	0	0
6	0.72	0.68	0.7	0.02828427	53000000	53000000	0	0	0	0	0	0
12	1.78	1.7	1.74	0.05656854	131000000	127000000	0	0	0	0	0	0
21	2.65	2.34	2.495	0.2192031	192000000	188000000	20	20	1.0417E-05	1.0638E-05	1.0527E-05	1.1082E-07
30	2.48	2.1	2.29	0.26870058	175000000	168000000	134000	115000	0.06979167	0.06117021	0.06548094	0.00431073
46	2.2	2	2.1	0.14142136	163000000	159000000	2450000	2600000	1.27604167	1.38297872	1.3295102	0.05346853
54	2.2	1.99	2.095	0.14849242	162000000	158000000	12000000	10500000	6.25	5.58510638	5.91755319	0.33244681
78	2	1.97	1.985	0.0212132	177000000	172000000	20500000	19000000	10.6770833	10.106383	10.3917332	0.28535018
102	1.9	1.77	1.835	0.09192388	140000000	133000000	25600000	24200000	13.3333333	12.8723404	13.1028369	0.23049645
126	1.44	1.23	1.335	0.14849242	98600000	97700000	28000000	27000000	14.5833333	14.3617021	14.4725177	0.1108156

Table S4. Small round cells formation data in $S7_{50}$

	Optical density (OD)		Mean of OD	Standard Standard deviation (SD) of OD	Total number of cells counted		Number of small round cells counted		% of small round cells		Mean % of small round cells	SD of small round cells
Time (h)	Exp. #1	Exp. #2			Exp. #1	Exp. #2	Exp. #1	Exp. #2	Exp. #1	Exp. #2		
0	0.05	0.05	0.05	0	550	600	0	0	0	0	0	0
6	0.2	0.21	0.205	0.00707107	800	800	0	0	0	0	0	0
12	0.68	0.72	0.7	0.02828427	500	550	0	0	0	0	0	0
21	1.25	1.33	1.29	0.05656854	600	550	1	1	0.16666667	0.18181818	0.17424242	0.01071374
30	1.69	1.74	1.715	0.03535534	500	700	1	2	0.2	0.28571429	0.24285714	0.06060915
46	2.69	2.72	2.705	0.0212132	800	950	8	10	1	1.05263158	1.02631579	0.03721615
54	3.01	3.1	3.055	0.06363961	800	900	9	10	1.125	1.11111111	1.11805556	0.00982093
78	3.3	3.7	3.5	0.28284271	950	800	65	53	6.84210526	6.625	6.73355263	0.1535166
102	2.1	2.3	2.2	0.14142136	850	900	132	134	15.5294118	14.8888889	15.2091503	0.45291807
126	1	1.4	1.2	0.28284271	900	800	129	116	14.3333333	14.5	14.4166667	0.11785113

Table S5. Small round cells formation data in LB

	Optical density (OD)		Mean of OD	Standard deviation (SD) of OD	Total number of cells counted		Number of small round cells counted		% of small round cells		Mean % of small round cells	SD of small round cells
Time (h)	Exp. #1	Exp. #2			Exp. #1	Exp. #2	Exp. #1	Exp. #2	Exp. #1	Exp. #2		
0	0.05	0.05	0.05	0	700	550	0	0	0	0	0	0
6	1.15	1.1	1.125	0.03535534	650	500	0	0	0	0	0	0
12	3.9	3.72	3.81	0.12727922	500	600	0	0	0	0	0	0
21	6.02	5.9	5.96	0.08485281	600	550	0	0	0	0	0	0

30	4.43	4.6	4.515	0.12020815	800	750	0	0	0	0	0	0
46	2.9	2.38	2.64	0.36769553	800	950	0	1	0	0.10526316	0.05263158	0.07443229
54	2.6	2.24	2.42	0.25455844	750	800	1	2	0.13333333	0.25	0.19166667	0.08249579
78	2	1.85	1.925	0.10606602	900	950	2	2	0.22222222	0.21052632	0.21637427	0.00827025
102	1.8	1.65	1.725	0.10606602	950	800	3	2	0.31578947	0.25	0.28289474	0.04652018
126	1.2	0.8	1	0.28284271	850	950	4	4	0.47058824	0.42105263	0.44582043	0.03502696

Table S6. Small round cells formation data in DSM

	Optical density (OD)		Mean of OD	Standard deviation (SD) of OD	Total number of cells counted		Number of small round cells counted		% of small round cells		Mean % of small round cells	SD of small round cells
Time (h)	Exp. #1	Exp. #2			Exp. #1	Exp. #2	Exp. #1	Exp. #2	Exp. #1	Exp. #2		
0	0.05	0.05	0.05	0	450	500	0	0	0	0	0	0
6	0.72	0.68	0.7	0.02828427	700	600	0	0	0	0	0	0
12	1.78	1.7	1.74	0.05656854	500	500	0	0	0	0	0	0
21	2.65	2.34	2.495	0.2192031	750	650	0	0	0	0	0	0
30	2.48	2.1	2.29	0.26870058	950	1200	0	1	0	0.08333333	0.04166667	0.05892557
46	2.2	2	2.1	0.14142136	1000	950	1	1	0.1	0.10526316	0.10263158	0.00372161
54	2.2	1.99	2.095	0.14849242	900	850	3	2	0.33333333	0.23529412	0.28431373	0.06932419
78	2	1.97	1.985	0.0212132	1050	950	5	4	0.47619048	0.42105263	0.44862155	0.03898834
102	1.9	1.77	1.835	0.09192388	950	900	5	5	0.52631579	0.55555556	0.54093567	0.02067564
126	1.44	1.23	1.335	0.14849242	850	950	8	8	0.94117647	0.84210526	0.89164087	0.07005392

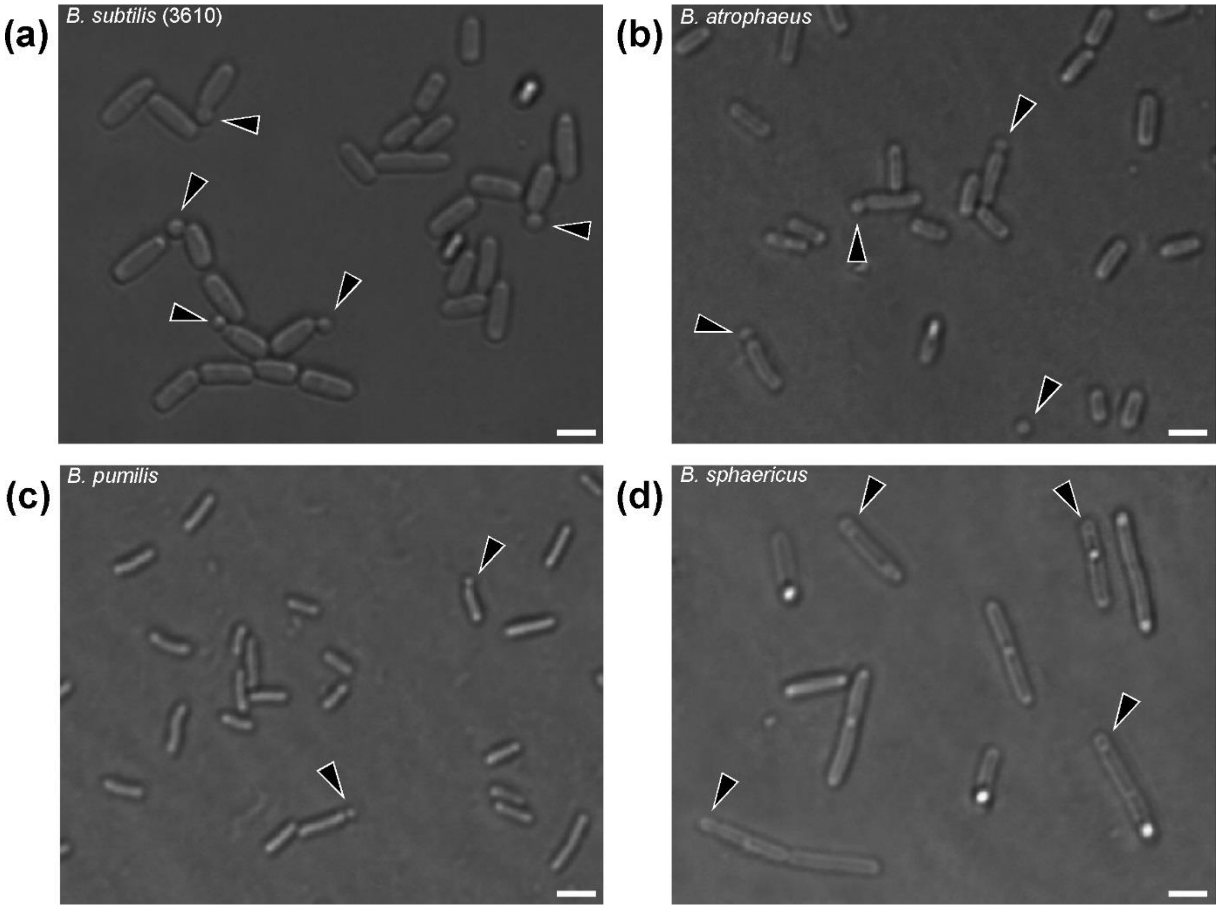

Fig. S1 Small round cells generation is not limited to *B. subtilis.* a) Undomesticated *B. subtilis*, b) *B. atrophaeus*, c) *B. pumilus* and d) *B. sphaericus* also produce small round cells. Note the particular characteristic of small cells generated by *B. sphaericus*; it appears that they do not round-up and seem to remain attached to their larger siblings. Cells were grown in $S7_{50}$ at room temperature. Black triangles indicate small round cells. Scale bars 2 µm.

(a)

(b)

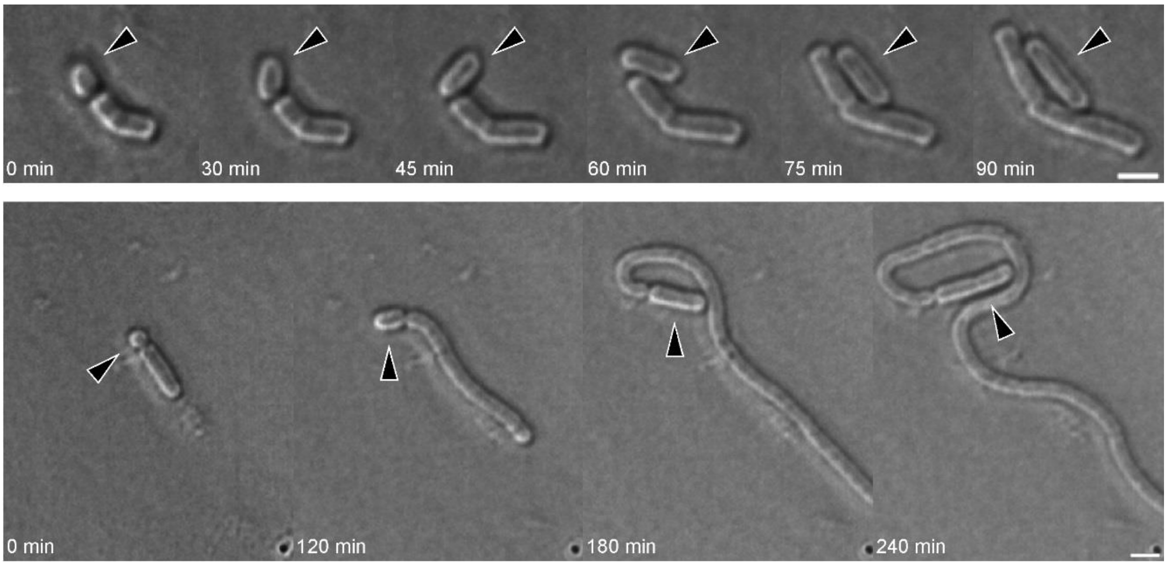

Fig. S2 Time-lapse microscopy of growing small round cells. Black triangles indicate small round cells that grow back to rod shape. Images were acquired every 15 min (a) and every 30 min for the first hour and every hour for the following 3 hours (b). Cultures of *B. subtilis* generating small round cells were diluted 1:10 into fresh growth media and grown for 60 min before time-lapse experiments. Scale bars 2 µm.

Movie S1 Time-lapse microscopy of growing small round cells (2 frames/s). The movie corresponds to the time-lapse experiments shown in Fig. 6a-c. Images were taken every 30 min.
